# A power law study of the edge influence on the perceived filling-in brightness magnitude

**DOI:** 10.1186/s41155-019-0130-7

**Published:** 2019-09-18

**Authors:** Marcelo Fernandes Costa, Carlo Martins Gaddi

**Affiliations:** 1 Departamento de Psicologia Experimental, Instituto de Psicologia, Universidade de São Paulo, São Paulo, Brazil; 2 Núcleo de Neurociências e Comportamento e Neurociências Aplicada, Universidade de São Paulo, São Paulo, Brazil

**Keywords:** Brightness perception, Brightness magnitude, Filling-in, Edge blur, Magnitude estimation, Spatial vision, Visual psychophysics

## Abstract

**Abstract:**

**Background:**

Edge plays a special role in spatial perception and as well as in determining the brightness of a surface within borders. The aim of our study was to measure threshold brightness in different levels of edges thickness.

**Methods:**

Steven’s power law for circles modulating in luminance was estimated for 30 subjects (mean age 24 years, SD 3.3, 13 female). Stimuli were presented on the iMac display using the 11-bit graphic board and consisted of two circles of 3° of visual angle, separated by 10°. We tested 7 levels of Michelson contrast: 7, 8, 10, 15, 26, 50, and 100. Three edges filtering were tested (0.3, 0.8, and 1.5° of smoothing). The subjects’ task was to judge the brightness of the edge filtered circle compared with the circle of the hard edge which was considered the modulus and received an arbitrary level of 50, representing the amount of brightness perception. In each trial, the same contrast level was presented in both circles. Five judgments were performed for each contrast level in edge filtering.

**Results:**

We found an increase in the power law exponent as the increase of the edge filtering (for sigma of 0.3 = 0.43, sigma of 0.8 = 0.73, and sigma 1.5 = 0.97). All power function fitting had high correlation coefficients (*r*^2^ = .94, *r*^2^ = .95, *r*^2^ = .97, respectively to sigma 0.3, 0.8, and 1.5) passing to the model’s adhesion criteria.

**Conclusions:**

There was a progressive distortion on the figure brightness perception as increasing the edge filtering suggesting the control of edges on the polarity of the overall brightness. Also, perceived brightness was increasingly veridical with increased filtering, approaching 1:1 correspondence at 1.5 sigmas.

## Introduction

Visual contours and surfaces are essential to our spatial perception. In continuation, they are related to the visual segmentation processes which underlie fundamental perceptual constructions like figure-background organization (Ghose & Palmer, 2010). Although many studies have addressed the topic, it is unclear if the surface brightness is a filling-in process, which is edge-dependent (Dakin & Bex, 2003; Vladusich, Lucassen, & Cornelissen, 2006), or if a brightness induction mechanism, based on the spatial filtering of the surroundings, would be a better predictor of the circle’s edges in association with being a function of their surroundings (Robinson & de Sa, 2013).

Physiological evidence shows that border-to-surface organization occurs at early stages of visual processes, and that they are related to interactions between cortical areas 17 and 18 (Hung, Ramsden, & Roe, 2007). This shows a large discrepancy in the temporal properties of induction suggesting that the indirect quadrature motion technique and direct brightness matching reveal different brightness induction mechanisms with different temporal characteristics (Blakeslee & McCourt, 2008). Considering the varieties of neurons in the early visual cortex, modeling their detection performance indicates the front-end filters underlying human edge detection are relatively small, consistent with receptive field sizes of simple cells in areas V1 and V2 (Elder & Sachs, 2004). Although, specific V1 cells weighing certain spatial frequencies with different numbers of cell filters they could be efficient to carry out edge detection filtering on an image (C. F. Stevens, 2015). On the other hand, recent studies using BOLD responses of fMRI in humans, have been shown that V3A and V4 cortical areas are selective to edges rather than lines. This occurs in both achromatic and equiluminant red-green patterns suggesting that dorsal and ventral pathways could be involved in form processing (Castaldi, Frijia, Montanaro, Tosetti, & Morrone, 2013).

The psychophysical task of location and detection of edge embedded in a brown noise has been used to localize optimal edge detectors, since it is possible to determine what observers are really looking for when they perform a visual task, in a more realistic and direct way (McIlhagga, 2018). Their results show a detection step of 0.1 to 0.17° of a Gaussian filter for edge detection, similar to receptive field sizes found in macaque V1.

Furthermore, it is well established that contour signals generate suppression of brightness in the center of a target (Paradiso & Nakayama, 1991) and the luminance contrast of the stimuli’s edge extends into the center. Also, lightness-darkness asymmetries are evident in bright-to-dark modulation (Paradiso & Hahn, 1996). In addition, darker areas at the edges of stimuli contribute to the darkening of the stimulus’ center whereas lighter areas at the edges further contribute to the brightening of the stimulus’ center. However, one question which was not addressed in those studies: What would be the perception of luminance on stimulus surface with a less evident border?

More recent edge integration models have proposed that the edge’s width in the computation depends on the contrasts of other nearby edges and on both the contrast polarity of the edge whose weight is being controlled as well as the polarities of other nearby edges (Rudd, 2013; Rudd & Zemach, 2004).

In our study, we measured the magnitude of brightness in circles by filtering with different levels of edge enabling us to elucidate how borders affect the subjective magnitude of the perceptual filling-in of surface brightness. Thus, as we increase the border filtering by increasing the blurring on the circle edges, we are aiming to verify if different filtering induces different brightness on the central part of the stimulus and, in continuation, if the power-law exponent fluctuates across filtering conditions.

## Methods

### Subjects

Thirty subjects (mean age = 24 years; SD = 3.3, 13 female) were binocularly tested. Subjects were recruited among the students and staff of the Institute of Psychology of the University of São Paulo, Brazil. Inclusion criteria included having best-corrected visual acuity of 20/20 or better measured monocularly at 4 meters using an ETDRS chart–tumbling E (Xenonio Rep. Prod., Sao Paulo, Brazil), refraction of ≤ 3.0 diopters considering the spherical equivalent of astigmatism values, absence of clinically evidence of ophthalmological diseases, and absence of known neurological and systemic diseases.

The study was approved by the Ethics Committee of the Institute of Psychology of the University of Sao Paulo and all the volunteers agreed to participate in the study after giving informed consent; yet, they were naive to the specific experimental question. Moreover, this study is also in accordance with the ethical standards laid down in the 1964 Declaration of Helsinki.

### Stimulus and equipment

All the stimuli psychophysical protocol was developed using Psykinematix v1.4.2, running on a computer iMAC OS with an integrated monitor of 21.5” and an NVIDIA GeForce GTX 675MX having 1.0 GB. The calibration of our experimental setup was performed for three aspects of the stimulus display: its geometry, its gamma function, and the color properties of its’ phosphors. The monitor was corrected for pixel geometry correcting the number of pixels to cm^2^. The gamma correction for the white and the three-color guns (red, green, and blue) was performed using a Minolta CS-100A (Konica Minolta Sensing Americas, USA). A total of 128 readings were performed for each color and for the white and the calibration fitting had an *r*^2^ > .97 for all guns. The colorimetric data was calibrated considering the CIE 1931 xy color space and the obtained formation feed the xy chromatic coordinates and maximum luminance (Lmax) for each phosphor which form the Maxwell triangle (or gamut) of the display. A bit-stealing procedure ensured the monitor resolution was at 10.8 bits (luminance steps 0.2%). The mean luminance of the monitor measured on a completely dark room was 74.03 cd/m^−2^.

**Fig. 1:**
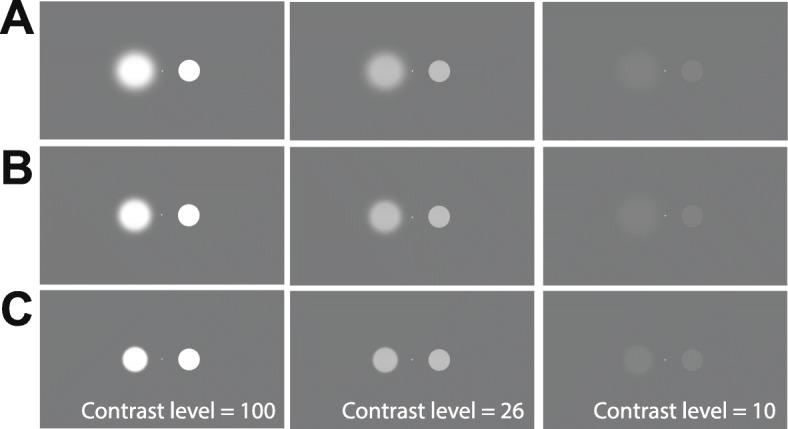
A sample of the subject’s viewing conditions. On the rows, we can see different levels of edge filtering in which **a** is the higher filtering (σ = 1.5), **b** is the medium filtering (σ = 0.8), and **c** is the lower filtering (σ = 0.3). From left to right columns are the samples of high, medium, and low luminance levels. The right circle with a sharped edge is the reference circle, and both circles had the same luminance

Stimuli consisted of two circles with a radius of 2° of visual angle at 60 cm of viewing distance, which was separated from each other by 15°. The stimuli were presented on a gray background of mean luminance (Fig. 1).

We tested 7 fixed levels of Michelson contrast: 7, 8, 10, 15, 26, 50, and 100 in three different edge filtering conditions 0.3, 0.8, and 1.5 sigma values (in degree) of a circular Gaussian. The higher the sigma value, the higher the edge blurring. The contrast level and edge filtering were presented in pseudorandom order. During the first 5 min prior to beginning the measurements, the eyes’ of the volunteers were adapted to the mean gray luminance. A black fixation dot was presented during the entire adaptation period. The pair of stimulus and modulus was presented for 1000 ms. As soon as there was a response from the subject, by pressing the space bar on the iMac keyboard, a new stimulus pair was presented. No time limit was defined. The values of the participant were recorded by the experimenter.

The reference (modulus) stimulus was at the same contrast level as the contrast being tested in each trial; however, it had a sharpened and well-defined edge (no edge blurring). For convenience, the modulus always appeared at the right side of the monitor while the test stimulus was located on the left side.

### Procedure

The participants were seated in a comfortable chair located 60 cm from the monitor screen and were oriented to keep their eye fixated on an insignificantly sized, black cross centered on the screen while blinking normally. No head stabilization device was used, and the eyes were leveled at the center of the monitor. Arbitrarily, the number 50 was attributed to the modulus’ brightness. In each trial, the participant’s task was to judge the central brightness of the circle with blurred edge, located at the left the side of the monitor, and were instructed to assign a number proportional to the perceived change in brightness related to the modulus’ brightness, found in the right side of the monitor. For example, if a stimulus appeared to have twice the brightness in comparison to the modulus, their value would be 100, respectively; if the brightness appeared to be one-fifth of the reference, the responding number would be near 10, and so on. All subjects were instructed to judge as precisely as they could include whole numbers as well as decimals. All subjects performed 10 judgments for each edge filtering and contrast level. The geometric mean was used to calculate the brightness judged for each subject. The test was performed binocularly in a darkened room and the total measurement was performed in approximately 30 min.

### Training session

Before the participants started the experiment, a line length judgment task was used as a training procedure. The procedure consisted of a series of 7 lines differing in sizes (ranging from .7 to 32 cm, presented in the center of a monitor screen. An additional line at 13 cm was used as the modulus and received the value of 50. This line remained visible throughout the experiment. The participant judged the line series two times. As soon as the power law adjustment showed a low dispersion rate (*R*^2^ > .90) and the exponent value was around 1.0 as suggested by Stevens (S. S. Stevens, 1956), we claimed that subject understood the task and could continue onto the experimental part. All participants needed 2 to 4 training sessions to get the criteria.

### Data analysis

The geometric mean of the 10 judgments was calculated for each participant. They were averaged and plotted in a log-log graphic. A power function was fitted on data according to Steven’s power law

$$ \varphi =k.{I}^n $$

in which the perceived brightness (φ) is a function of the stimulus intensity (*I*) powered by the exponent (*n*), corrected by a constant (*k*). The exponent of 1 means there is a proportional change between the stimuli and the perceptual continuums. Exponents higher than 1 represent higher changes in perception in comparison towards the stimuli. In vice versa, exponents lower than 1 show the opposite: higher changes in stimuli in comparison to the perception.

Data analysis was performed in Statistica v 10.2 (StatSoft, Tulsa, USA) software pack for statistics. This included the geometric mean calculation, averaging, and fitting procedure. A one-way ANOVA was used to calculate significant differences between the exponents, and the Tukey post hoc test was used to analyze which groups were different.

## Results

**Fig. 2:**
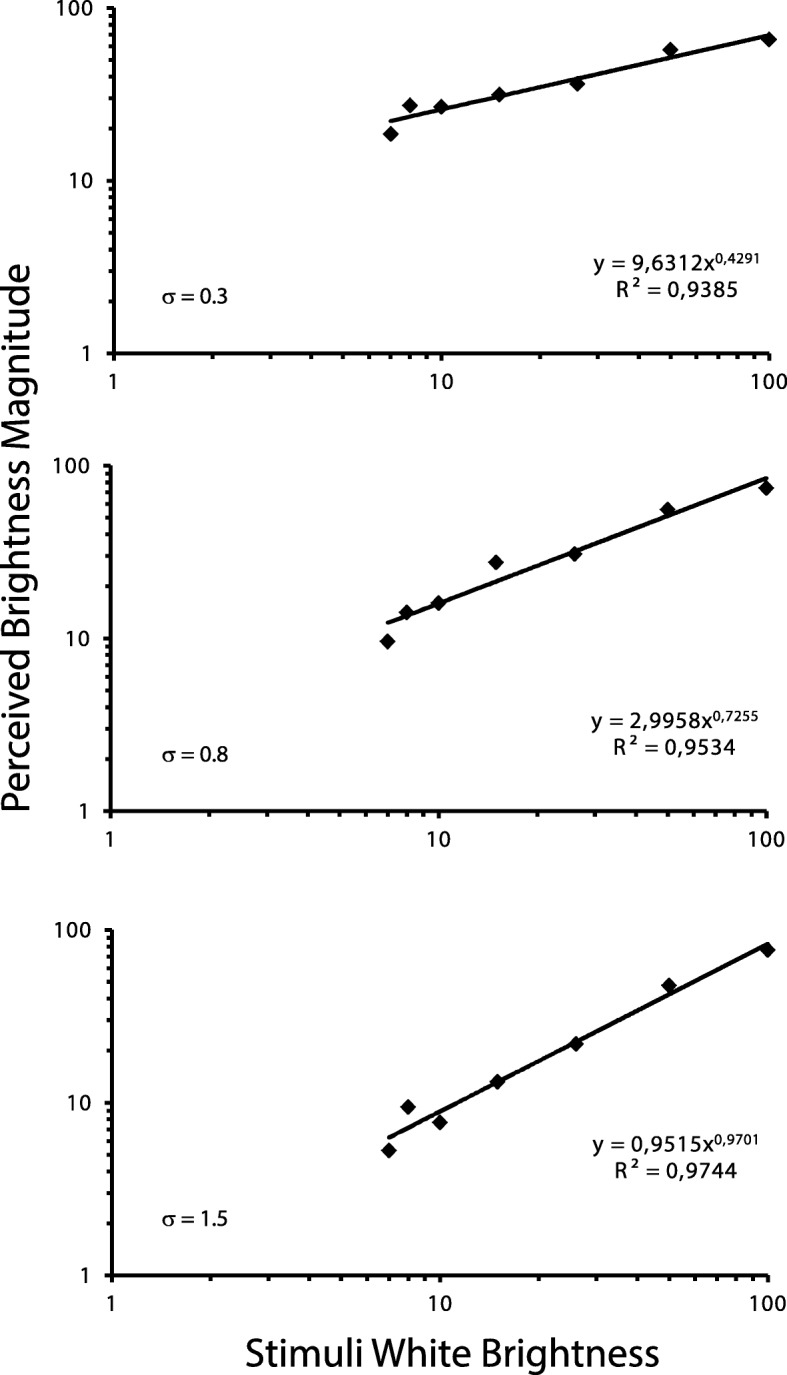
Brightness magnitude perceived for circles with different contrast levels in 3 edge filtering conditions (σ = 0.3; σ = 0.8; σ = 1.5). As the filtering increases, the higher the exponent changes from 0.43 to 0.73 and to 0.97, respectively. The increasing on the power law exponent indicates the weakening of the edge influence on the brightness of the circle

The magnitude estimated for the brightness was displayed in a function incorporating the stimulus contrast. We found an increase in the power law’s exponent also showed an increase the filtering. For filtering sigma of 0.3, the exponent was 0.43; for filtering sigma of 0.8, the exponent was 0.73; and for filtering sigma of 1.5, the exponent was 0.97) (Fig. 2).

**Fig. 3:**
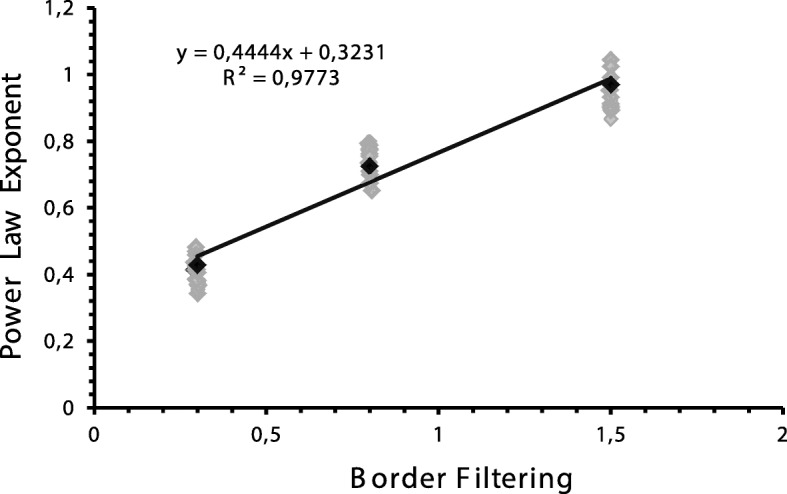
A linear increasing on the power law’s exponent, as a function of the amount of the edges’ filtering suggests a proportional subjective impression of the brightness with the increasing of the edge blur

There was a significant difference between the power function’s exponents (*F* = 3.17; *p* = .003) and the three groups as they differed. When comparing the function of the power law’s exponents against the sigma values, which generated the border filtering, we found a well-adjusted linear function highlighting the increase of the brightness perception (Fig. 3).

## Discussion

We found that the magnitude of the perceptual filling-in for the surface brightness was strongly affected by the edge filtering. As the filter increased, escalating the edge’s blurriness, the brightness for the central region for that circle also increased in a power function fashion. We could interpret it as a weakening of the edge influence on the surface’s brightness. The lower exponent (0.43) at 0.3 sigmas indicated a compressive brightness judgment—specifically an increase in the brightness judgment for lower luminance levels. This compression disappeared with greater smoothing, and at 1.5 sigmas the exponent is 0.97, reflecting a 1:1 match in the brightness judgments between the test conditions.

In our experiment, as we filtered the edges, we progressively eliminated the local, high-spatial frequencies as we increased the blurring values. The perceived brightness became more proportional to the lightness as the edge filtering increased. Since the filtering removes high spatial frequencies, the increase in the central region of the stimuli could be mediated predominantly by low spatial frequencies. In previous studies, similar conclusions have been reached in which luminance Chevreul-staircases were filtered for spatial frequencies (Peromaa & Laurinen, 2004). The authors found that only low spatial frequency components of edges were able to trigger brightness filling-in. In addition, they stated high spatial frequency components of edges do not produce a perceived brightness but only featured characteristics as lines. According to Perna and Moronne, the high-spatial-frequency filtering above 2 c/deg was never sufficient to raise the perceived difference of brightness above the threshold. Thus, the mechanism tuned to frequencies lower than 1 c/deg could mediate the filling-in brightness perceived. That is the case of our study, in which the stimuli were of .50 c/deg and lower as the filtering increased. Additionally, our results provide complementary information on brightness perception since evidence reported changes in the amount of brightness perceived as the edge filtering occurred. Future measurements using smaller and larger sigma should be performed to better comparisons with literature data.

Edges are also important for spatial location. In an alignment task targeting the changes in the blurriness of the edges of white bars, there was a consistent shift on edge location as it varied according to the level of stimulus’ contrast (Bex & Edgar, 1996). For well-defined edges, an increase in contrast did not change the alignment position; however, as the edge blurring increased, for raising contrast levels, there was also an increase in the shifting of the white stripes in a direction to the adjacent black region. It seems that with an increase in the contrast level as well as the edge blurring, there was an expansion of the total area perceived, which, in turn, shifted our perceptual location for the edges.

In some sense, our data complements those findings, since the blurriness of our stimuli generates an increase in the contrast perceived. One possible hypothesis for the contrast increasing could be related to the size enlargement. As we blurred the edges, there was an apparent increase in the apparent size of the circle. A possible mechanism which could compensate for the increase in size could be an increase in the contrast level. Indeed, our data could provide complementary information for the findings of Bex and Edgar (Bex & Edgar, 1996). Blurring the edges generated an expansion in the total area perceived, a consequent change in the edge location*,* and a compensatory increase in the perceived brightness. In our case, an additional effect was observed because the increase was polarity dependent, due to the fact that the bright grays appeared brighter and the dark grays appeared darker. Future measurements using multiple circle sizes with constant relative-size filtering would be a more appropriate approach to this question. Although, large sizes stimuli have been employed in different conditions than those we tested, it can give us support for hypotheses of what to expect from these future experiments. Recent studies using spatial complex stimuli in which contrast edges were embedded in a brown noise (1/f^2^) found that an edge detector profile with a peak to trough width of approx. 0.1 to 0.17° of difference between adjacent areas was needed for an edge to be detected (McIlhagga, 2018). Since authors used stimulus comprised of10° of visual angle, we could argue that an increase in size by changing the overall brightness altering the power law exponent could not be supported.

A consequence of the bright stimulus is that it becomes subjectively brighter; in addition, the dark stimulus is perceived as darker with the increase of the power law’s exponent. This increase in the contrast shifting relating it back to the edge blur affected the subjective contrast range, and, as a consequence, also increased in the exponent measured. Experimental evidence for the changes in the exponent regarding the range of the stimuli can be found in several studies (Lockhead & Hinson, 1986; Pradhan & Hoffman, 1963; Teghtsoonian, 1973). Our results provide support for the causal relation between our perceptual mechanisms and the range of the stimuli managing the edge filtering. A practical outcome of the brightness reduction to reach higher ranges is associated with the impairment in eyes’ saccadic movements, which reduces significantly the visual search precision (Gilchrist, Humphreys, Riddoch, & Neumann, 1997). In addition, as well as in the binocularity since there is an increase in the cortical disparity computation (Georgeson & Wallis, 2014).

The power law’s exponent was around 1.0 measured for the blurriness of 1.5 Gaussian sigma. This means that the magnitude of perceived brightness changed proportionally to the central region of the circle brightness and, thus, led to another important consequence of the phenomena, there were no edges’ influences on brightness perceived. These results suggest that surface brightness is a filling-in process more than an induction mechanism. As one increases the blurring of the edges, there is a perceptual shift of the edge to regions farther from the center of the circle. This displacement weakens the edge effect on the center which reduces its’ filling-in effect. Since the background was constant, the brightness induction remains the same for all the filtering conditions. Reducing the filtering enlarges the edge, and, consequently, the transition distance between the center of the circle and the surrounding would reduce as well—ultimately, the expected effect would be the opposite.

It is a worthwhile and open question as to why edge integration has evolved to be the favorite physiological mechanism to generate a representation of surface information. In addition, our study contributes to understanding an explanation of human blur vision. As we move away from the optimal edge detection condition, we have an exponential reduction of the brightness perception of spatial adjacent areas with a reduction in contrast detection and, consequently, in our spatial vision. Increases in blurring reduce the high structural complexity of visual scenes, decreases detection of high spatial frequencies, and negatively impacts local feature vision. As a direct consequence, spatial discrimination is reduced, negatively impacting elementary perceptual functions as a figure-ground organization (Ghose & Palmer, 2010).

This study used only three edge filters. It started with an edge detector size that was twice the limit found in previous studies, because we were interested in the brightness changes for different suprathreshold edge filtering, which could be a limitation of this study. Future investigations considering the transition between optimal edge filtering size 0.1–0.17 and ours, which started filtering at 0.30, would be important to model how brightness perception behaviors in this filtering range function. Also, future studies could integrate the findings of our work to improve computational modeling of visual neurons (Yedjour, Meftah, Lezoray, & Benyettou, 2017).

## Conclusions

We concluded that the brightness perception at the center of the stimuli receives interferences from the high spatial frequencies of the edges. This is because the filtering of high spatial frequency in blurring the edges changed the brightness perceived to values closer to the physical stimuli. Our results suggest that edges reduce the range of the brightness perception of the stimuli as a whole. However, it is not clear, how the brightness in central regions dominated by low spatial frequency channels could be affected by the high spatial frequencies presented at the edges.

## Data Availability

The datasets used in the current study are available from the corresponding author. Please contact the corresponding author for data requests.
